# Achieving Consensus in the Development of an Online Intervention Designed to Effectively Support Midwives in Work-Related Psychological Distress: Protocol for a Delphi Study

**DOI:** 10.2196/resprot.4766

**Published:** 2015-09-04

**Authors:** Sally Pezaro, Wendy Clyne

**Affiliations:** ^1^ Centre for Technology Enabled Health Research Faculty of Health and Life Sciences Coventry University Coventry United Kingdom

**Keywords:** Delphi technique, Internet, intervention studies, midwifery, psychological, research protocols, self-help groups, stress

## Abstract

**Background:**

The development of an online intervention designed to effectively support midwives in work-related psychological distress will be challenging due to the ethical, practical, and therapeutic issues surrounding its design. Related literature suggests that midwives may require an anonymous, confidential, and therapeutic platform that facilitates amnesty and nonpunitive approaches to remedy ill health. However, it is unclear which requirements may be most salient to midwifery populations.

**Objective:**

The objective of this paper is to describe the design of a Delphi study, intended to achieve expert consensus on the needs of midwives in work-related psychological distress who may be supported via an online intervention. This protocol may also serve as a research framework for similar studies to be modeled upon.

**Methods:**

A heterogeneous sample of at least thirty experts on psychological well-being and distress associated with midwifery work will be recruited. Their opinions regarding the development of an online intervention designed to support midwives in work-related psychological distress will be collected through 2 rounds of questioning, via the Delphi Technique. When 60% (≥18, assuming the minimum is 30) of panelists score within 2 adjacent points on a 7-point scale, consensus will be acknowledged. This Delphi study protocol will invite both qualitative and quantitative outcomes.

**Results:**

This study is currently in development. It is financially supported by a full-time scholarship at the Centre for Technology Enabled Health Research at Coventry University (Coventry, UK). The implementation of this Delphi study is anticipated to occur during the autumn of 2015.

**Conclusions:**

The results of this study will direct the development of an online intervention designed to support midwives in work-related psychological distress, summarize expert driven consensus, and direct future research.

##  Introduction

### Background

The mental health and well-being of health care professionals has gathered significant attention due to its direct correlation with quality patient care [1]. Midwives may be at an increased risk of developing psychological distress due to the traumatic work environments they endure [2]. These environments report incidents of workplace bullying, emotionally demanding clinical case loads, and a pressure to work despite feeling unwell enough to do so [1,2]. Interventions designed to support midwives in work-related psychological distress are required if the global shortage of midwives and the poor effects that midwives’ psychological distress has on patient care are to be remedied. It is unclear who may be responsible for the well-being of health care staff in the United Kingdom, yet it is clear that there is a paucity of support for midwives in distress [3].

Midwives generally find it challenging to disclose personal experiences of psychological distress [4,5]. In addition, health care professionals who experience the distressing effects of functioning within traumatic work environments may not recognize mental ill health in themselves [6,7].

To enable midwives to seek help with the consequences of work-related psychological distress, a platform of amnesty, confidentiality, and anonymity may be required before any benefits may accrue [8,9].

### Can Online Interventions Be the Answer?

An online intervention may be one solution that midwives may turn to in work-related psychological distress, as a preferred option of support [10]. To develop an online intervention that fits the needs of midwives, their employers, and professional bodies, it will be important to first define what characteristics an online intervention should have.

This paper outlines a protocol for a Delphi study designed to achieve expert consensus about what midwives in work-related psychological distress may need to be supported via an online intervention and peer support platform. The expert consensus will be used to inform the development and content of an online intervention for midwives in work-related psychological distress.

##  Methods

### The Delphi Study Design

The Delphi Technique has been used extensively within health, social science, and intervention research [11-13]. It involves rounds of discussion whereby experts are invited to disclose their opinions on particular topics for which there is a paucity of knowledge. It is assumed that the opinions of many outweigh those of the individual, and thus, any consensus generated may be considered to be a valid expert opinion [14,15]. Because there is an incomplete state of knowledge about what midwives in work-related psychological distress may require when accessing an online intervention designed to effectively support them, a Delphi study was considered to be a suitable research tool to augment unanimity in opinion [16]. The distinct characteristics of the Delphi technique are (1)anonymity, (2) iteration, (3) controlled feedback, and (4) statistical “group response” [17].

Achieving consensus is the primary aim of the Delphi study, yet the measurement of consensus varies greatly [18]. There is no firm consensus as to what may be considered a consensus within a Delphi study. Within this Delphi study, a primary criterion is that at least 60% (≥18, assuming the minimum is 30) of Delphi panel members must indicate a preference within 2 adjacent response points on a7-point Likert scale for consensus to be reached.

Rigid Delphi study designs have been criticized for their inability to allow their experts to elaborate on their opinions [12]. Therefore, this Delphi design will be a modified one [15,19]. Free text response options will accompany each statement put to panel members [20] to provide experts with the opportunity to elaborate on their opinions.

The research team who will conduct this Delphi study includes 6 academics with professional backgrounds in midwifery, general medicine, psychology, and academic research.

### Participants

**Figure 1:**
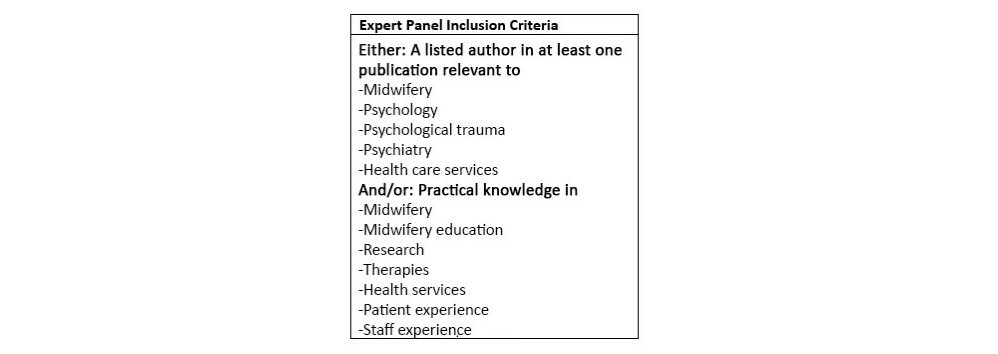
Participant inclusion criteria.

There are no clear guidelines in relation to what panel size is most appropriate for a Delphi study design [20]. A minimum of 30 experts will be recruited to this Delphi panel. Heterogeneity within the expert panel will play an essential part in ensuring study quality [16]. Therefore, panel members will be selected from different fields relating to midwifery care, health care, psychological distress, professional practice, and academia. They will be identified through a stakeholder analysis (see Multimedia Appendix 1). These experts will be midwives, researchers, lecturers, health care professionals, students, patient groups, and maternity-based organizations. Inclusion criteria are shown in Figure 1.

Once experts have been identified, they will be directed toward information about the aim and content of the Delphi study. A formal invitation will also be given (see Multimedia Appendix 2 and [21-31]). Potential participants will be invited to consent to participate as the online Delphi study begins (see Multimedia Appendix 3). Potential and recruited panel members will also be asked to refer other suitable individuals. This layer of recruitment aims to eliminate any bias from the research teams’ recruitment selection. Solicitation of nominations of appropriate field experts is typically recommended as best practice in the Delphi study design [32].

Informed consent will be obtained from all participants as the first round of questioning begins online, and will include the consensual agreement to publish anonymized data and nonidentifiable data results (see Multimedia Appendix 3). Participants will be directed to appropriate support services both online and offline due to the sensitive nature of the subject matter. Participants will also receive copies of any publications that may result from the study and a summary of outcomes.

### Participant Recruitment

#### Overview

**Figure 2:**
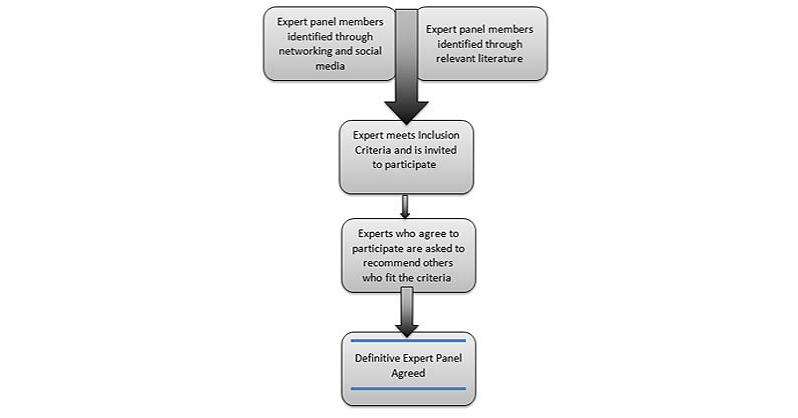
Flowchart for participant recruitment.

Experts will be invited to participate by the research team. They will be invited via email and social media contact with a formal invitation to become a part of the panel (see Multimedia Appendix 2). Figure 2 shows the flowchart for participant recruitment.

It is anticipated that some experts may withdraw from the study during its course [33]. Therefore, social media will also be used to recruit participants to compensate for potential dropouts. A minimum of 30 panel members will be recruited to this study, although the team recognizes that there is no consensus regarding what the optimal number of participants for a Delphi study may be [14,34]. Should less than 50 experts be recruited before the Delphi study commences, an additional 50 people will be invited to participate to compensate for potential dropout rates and to avoid a failure to achieve adequate panel numbers.

#### Social Networking Recruitment

The research team will consult their social, academic, and occupational networks to identify potential experts who meet the inclusion criteria. Suitable candidates will receive an email inviting them to participate in the Delphi study.

Twitter will also be used for research recruitment due to its high-quality health care, research, and academic communities. Twitter is evidenced to be a highly effective tool for health care research recruitment [35]. Stakeholder groups identified in the stakeholder analysis will then be asked to promote the study to their online followers. A link to a blog page with inclusion criteria, further information, support resources, and an online survey will be provided to facilitate online recruitment [36]. Willing and suitable participants can then express their interest in partaking in the study by contacting the research team directly.

#### Recruitment Through the Academic Literature

Experts within the field of midwifery, psychology, psychiatry, and health care will be identified through literature searching. The research team will identify key papers of relevance, the authors of which will then be invited to participate. They will be invited via email and social media contact with a formal invitation to become a part of the panel (see Multimedia Appendix 2).

### Procedure

#### Overview

This Delphi study will employ the principles of anonymity, repetitions at each stage of questioning, and feedback between rounds of descriptive statistics regarding the group’s response and summaries of free text responses about each item in the item panel [37]. The Delphi study technique was chosen as it prevents dominant individuals from controlling the process of group discussion [16]. This is particularly salient in hierarchal environments, such as the health care system, where many participants are anticipated to originate. The anonymity the Delphi study facilitates can also allow for unashamed freedom of speech, which in turn, leads to a more accurate opinion giving [38].

Experts will only be sent further correspondence should they indicate an initial interest to participate in the study. In the absence of any response to the initial invitation sent by the research team, it will be assumed that the recipient has no interest in participating in the study, and will therefore receive no further correspondence.

Experts who continue to participate within the study but do not respond to the first Delphi round will be sent 2 reminders via email or social media contact. To withdraw from the study, experts must directly contact the research team and explicitly state their withdrawal. Unless this action is confirmed, all experts will receive reminders and survey links for each round. Two weeks will be allocated for Delphi experts to respond to each round of questioning [39]. In total, there will be a 5-week interval between the initiation of the first round and the start of the second round of questioning.

Reminders will be sent to participants 1 week before each round begins in order to maximize their participation. A link to the survey will then be given to all participants.

#### Questions

Questions have been designed to explore consensus about the design, construction, purpose, and content of an online intervention to support midwives in work-related psychological distress (see Multimedia Appendix 4). These questions were developed in response to a review of the literature. This is an acceptable and a common modification of the Delphi process [39]. Literature reviewing remained broad in scope and included a combination of the search terms “burnout,” “psychological distress,” “midwives,” “midwifery,”“midwife,” “online intervention,” “self-help groups,” “CBT,” “mindfulness,” “stress,” “depression,” “anxiety,” “peer support,” “mental health literacy,” “second victim,” “PTSD,” “post-traumatic stress,” “workplace bullying,” and “NHS.” In reading and re-reading the retrieved literature, a theoretical basis was developed for what may or may not be useful in the development of an online intervention designed to support midwives in work-related psychological distress. These theories are put forward for testing before the expert panel.

There will be 3 themes of questioning and 2 response options available. The 3 themes will be intervention design and practical inclusions, inclusions of therapeutic support, and ethical inclusions. The 2 response options available will be a 7-Point Likert scale and open text responses.

### Delphi Survey Design

Bristol Online Survey [40] will be used to administer the Delphi study. Round 1 will consist of a structured questionnaire. Respondents will be asked to indicate their priority rating for a series of items via Likert scale responses. They will also have the option to disclose why they chose to mark each item with lower or higher priority within an open text field. Respondents will also be invited to provide additional comments through the provision of a free text response. Finally, panelists will have the opportunity to suggest new questions to be put forward during the second round of questioning.

Round 2 will consist of a second questionnaire that is based on the information provided in the first round. The primary aim of this round will be to offer the panel the opportunity to reconsider their responses from Round 1 for those items for which consensus was not achieved in Round 1. This opportunity will be offered in light of feedback about the groups’ responses in Round 1. New questions may also be added to this second round in response to suggestions put forward by the panel during the first round. Respondents will be asked to review these new questions and indicate their priority rating. Respondents will be invited to provide comments through the provision of a free text response option for each item in the second questionnaire. They will also again be given the opportunity to disclose why they have chosen to mark each item with lower or higher priority within an open text field.

### Analysis

Because there are no conclusive guidelines for establishing consensus in Delphi literature [41], taking account of the average accord and the 7-point scale, consensus will be reached if 60% (≥18, assuming the minimum is 30) of respondents are within 2 adjacent response points on the 7-point scale (eg, if 60%, ≥18 assuming the minimum is 30, of participants select 2 and 3 in response to a specific item). Items which do not achieve consensus in Round 1 will be re-presented in Round 2.

The mean, minimum, and maximum scores for each item will also be calculated and reported to panel members as feedback after each round.

Any free text responses provided by participants to specific items will be analyzed with thematic analysis [42]. Themes may be reframed, reviewed, and revised throughout this thematic analysis, as coherent patterns are formed. This thematic analysis of qualitative open responses will be presented in a table format and feedback will be provided to panel members after each round.

## Results

This study is currently in development. It is financially supported by a full-time scholarship at the Centre for Technology Enabled Health Research at Coventry University (Coventry, UK). Ethical approval for this study has been granted by Coventry University Ethics Department. The implementation of this Delphi study is anticipated to occur during the autumn of 2015. Project reference id P35069.

## Discussion

### Preliminary Agenda

The aim of this Delphi study is to reach consensus on the salient themes and elements to be included within an online intervention to support midwives in work-related psychological distress.The results of this research will be used to inform the development of an online intervention designed to support midwives in psychological distress.

A key weakness of the Delphi technique is that it lacks a theoretical framework [14]. The advantage of using a Delphi study technique within this research will be that ideas, definitions, and experiences of a variety of experts can be synthesized to inform development of the intervention. Panel members will be drawn from a variety of backgrounds, and as such will be able to contribute a variety of evidence and multidisciplinary perspectives.

Biases may occur in Delphi studies that could also distort the consensus. Desirability bias from both the experts and the research team could impede the achievement of a “true”consensus [43]. There is also the risk of ambiguity and conditional statements given within thequestionnaire [44]. In this case, panel members may be interpreting the questions and statements differently. This may also lead to a polarization in results. A 7-point judgment scale is used to avoid elements of ambiguity; however, this may not protect results against some polarity [45]. To mitigate these risks, the questionnaire has been reviewed and piloted among peers.

### Conclusions

This paper describes the design of a Delphi study. This will be the first Delphi study to explore the online support needs of midwives in work-related psychological distress.

## Appendix

Multimedia Appendix 1 Stakeholder analysis.

Multimedia Appendix 2 Formal invitation presented to potential participants.

Multimedia Appendix 3 Informed consent form.

Multimedia Appendix 4 Delphi study questionnaire.
