# Supplementary material for: Achieving Consensus in the Development of an Online Intervention Designed to Effectively Support Midwives in Work-Related Psychological Distress: Protocol for a Delphi Study
Source: JMIR Res Protoc. 2015 Sep 4;4(3):e107. doi: 10.2196/resprot.4766 (PMC4704889; doi:10.2196/resprot.4766)
Supplement: Multimedia Appendix 1 [file resprot_v4i3e107_app1.pdf]

Appendix 1: Stakeholder Analysis

Stakeholder (s)	Area of interest	Contribution	Expectation
Current government<br>CQC<br>DoH<br>CNST<br>NHSLA<br>NHS England<br>NHS Employers<br>NHS Wellbeing teams	• Improved NHS Services<br>• Government Policy<br>• Financial Savings<br>• Care Quality<br>• Reduced Risk/Litigation within NHS services.<br>• Good Staff Health<br>• Positive Patient Experience<br>• Good Recruitment and Retention Rates<br>• Targets<br>• Positive Public Opinion<br>• Risk Management<br>• Austerity Measures	• Policy Backing<br>• Low Risk Strategies for Implementation<br>• Leadership in Innovation<br>• A Clear Vision of Future NHS services<br>• Integration with Current Healthcare Guidelines<br>• Governance Standards for Consideration<br>• Quality Standard Setting and Risk Assessments<br>• Staff Survey Feedback<br>• Ongoing Audit of Outcome Measures	• Improvements in NHS Services<br>• Financial Savings<br>• Implementation of Government Standards/Vision/Guidelines and Overall Policies.<br>• Service Quality Assurance<br>• Compliance with Government Leadership Direction.<br>• CNST/CQC Standards Met<br>• Improved Staff Wellbeing<br>• Improved Recruitment and Retention Rates<br>• Evidenced Based Solution to poor NHS Staff Health.
NHS Trusts<br>Occupational health teams<br>Human resources departments	• Patient Satisfaction<br>• Increased Care Quality<br>• Staff Health<br>• Service Improvement<br>• Clinical Governance<br>• High CNST Level Attainment<br>• Risk/Litigation Reduction<br>• Staff Wellbeing<br>• Good Recruitment and Retention Rates	• Staff Survey Knowledge<br>• Motivation for Improving Care Quality<br>• Priority Setting<br>• Knowledge in Innovation<br>• Feedback<br>• Recommendations<br>• Wellbeing Strategies<br>• Current Guidance<br>• Policy Recommendations<br>• Ongoing Audit of Outcome Measures	• Service/Quality Improvements<br>• Financial Savings<br>• Reduced Risk and Litigation<br>• High CNST Level Attainment<br>• Quality Project Management<br>• Improved Reputation<br>• Reputation of 'Good Employer'<br>• Evidenced Based Solution to Poor NHS Staff Health

	• Good Reputation<br>• Targets		
Royal College of Midwives	• Midwife Wellbeing<br>• Staff Support<br>• Staff Representation<br>• Reputation of Profession	• Endorsement of Intervention<br>• Support for Users<br>• Positive Promotion of Initiative<br>• Feedback<br>• Contribute Recommendations<br>• Ongoing Audits of Outcome	• Improved Midwife wellbeing<br>• Evidenced Based Solution to Poor Staff Health<br>• Staff Support<br>• Uphold the Reputation of Midwives<br>• Represent Midwives Effectively
Nursing and Midwifery Council	• Public Safety<br>• Public Image<br>• Midwifery Regulation<br>• Midwifery Reputation	• Support for Project<br>• NMC Code Guidance<br>• Ethical Guidance<br>• Agreed Amnesty?<br>• Public Safety Guidance<br>• Feedback<br>• Contribute recommendations<br>• Audit Ongoing Outcomes<br>• Awareness	• Competent Registrants<br>• Healthy Registrants<br>• Reduction in Referrals<br>• Public Safety<br>• Improved Care<br>• Professional Reputation Maintained.
Matrons and Midwifery Leaders	• Maternity Service Improvement<br>• Staff Wellbeing<br>• Staff Morale<br>• 'Working Smarter'<br>• Reputation<br>• Targets<br>• Service Quality	• Staff Knowledge<br>• Knowledge to Promote Supportive Cultures<br>• Feedback<br>• Contribute Recommendations<br>• Audit Ongoing Outcomes<br>• Generate Awareness	• Improved Maternity Service Outcomes<br>• Overall Financial Savings<br>• Risk Reduction<br>• Improved Care<br>• Improved Staff Wellbeing<br>• Improved Staff Morale

Midwives and Student Midwives	• Self-Management<br>• Wellbeing<br>• Employment<br>• Competence<br>• Continued Professional Development<br>• Positive Working Cultures<br>• Effective Appraisals<br>• Effective Working Teams<br>• Quality Care Services<br>• Staff Wellbeing<br>• Good Reputation<br>• Targets Met<br>• Leadership	• Protected staff time<br>• Permission for Project to go ahead/Continue<br>• Allocation of Resources<br>• Supportive Culture<br>• Feedback<br>• Contribute recommendations<br>• Project Support<br>• Generate Awareness	• Improved Maternity Service<br>• Overall Financial Savings<br>• Risk Reduction<br>• Improved:<br>• Care<br>• Staff Wellbeing<br>• Morale<br>• Good Health<br>• Supportive Employers<br>• Supportive Regulatory Bodies<br>• Supportive Representative Bodies
Service users and the public	• Improved Maternity Services<br>• Care Experience<br>• Reduced Risk<br>• Good Outcomes<br>• Professional Reputation<br>• Maintain Trust<br>• Midwife Wellbeing	• Patient and Public Opinion on Midwife Working Cultures<br>• Opinions upon Ethical Dilemmas of Amnesty.<br>• Public Expectations<br>• Awareness<br>• Support	• Public Safety<br>• Service Improvements<br>• Reduced Risk<br>• Positive Outcomes<br>• Healthy Midwives
