# Supplementary material for: Achieving Consensus in the Development of an Online Intervention Designed to Effectively Support Midwives in Work-Related Psychological Distress: Protocol for a Delphi Study
Source: JMIR Res Protoc. 2015 Sep 4;4(3):e107. doi: 10.2196/resprot.4766 (PMC4704889; doi:10.2196/resprot.4766)
Supplement: Multimedia Appendix 2 [file resprot_v4i3e107_app2.pdf]

Appendix 2: Formal invitation to be sent to potential participants - Participant Information Sheet

Formal Invitation to participate in an online Delphi Study to achieve Consensus in the Development of an Online Intervention Designed to Effectively Support Midwives in Work related Psychological Distress.

Traumatic work environments in maternity services may be associated with stress, vicarious trauma and anxiety (36), (37), (9), (38). The need for an evidenced-based intervention is pressing, as healthcare staff report that they are close to having a breakdown in mental health (39).

Currently, there is a paucity of support for midwives, who could be at an increased risk of psychological distress due to the fact that they are independent practitioners, working in an area of high litigation (3), (40), (41). NHS staff surveys highlight workplace cultures that are not midwife-friendly (40). This is of significance, as there is a clear link between staff wellbeing and patient care (42), (43), (44) (45), (1).

Online networks are one option people may turn to in psychological distress. As a consequence, midwives may find online networks a place to acquire the skills and resources required to manage their own mental wellbeing, improve their professional quality of life, and reduce symptoms of traumatic stress, depression and anxiety (46).

The focus of this research is to reach expert consensus on the needs of midwives in work-related psychological distress who may be supported via an online intervention. We are looking for your opinion, as part of an anonymous expert panel, in regards the development of an online intervention designed to support midwives in work-related psychological distress. We invite you to participate in 2 rounds of questioning, which explore what may be prioritized within such an online intervention.

We hope that the results of this study will form a consensus in how an intervention designed to support midwives in psychological distress should be designed, developed and used. The results of this study will direct the development of an online intervention designed to support midwives in work-related psychological distress, summarise expert driven consensus and direct future research.

You have been invited to participate in this study due to your expert knowledge in one or more of the following areas:

- Expert knowledge in Midwifery
- Psychology
- Psychiatry
- Psychological Trauma
- Healthcare services
- Practical knowledge in Midwifery
- Midwifery Lecturing
- Research
- Therapies
- Health Services
- Patient experience
- Staff Experience

However, you have no obligation to take part in this study and may decline or withdraw from the study at any time without need of any explanation.

Should you wish to take part, please express your interest in participation to the primary researcher, Sally Pezaro via the contact details listed below.

The first round of questioning is anticipated to take place during the first 2 weeks of October 2015. You will be asked to give your formal informed consent to participate at the beginning of the online questionnaire. This questionnaire should take around 30 minutes to complete, however you will have the opportunity to expand upon your answers to your own extent. You will also have the opportunity to suggest further questions to be put forward to the panel. You will be given 2 weeks to complete and submit your considered responses. You may also receive reminders to complete the survey should you need prompting to do so.

The research team will collate and evaluate all responses. The team will then produce a report which sets out all responses and results of the study. We anticipate that there will be some questions which may or may not reach group consensus. When 60% of panelists score within 2 adjacent points on a 7 point scale, consensus will be acknowledged. This will also be reported. You will receive a copy of the full report.

Three weeks after the submission deadline for the first questionnaire, you will be sent a link to a second online questionnaire. This questionnaire will contain

questions that may not have achieved consensus during the first round of questioning. You will be asked to review your initial response and be given the opportunity to amend your initial response should you wish to, based upon the overall response reported from the entire panel. There may also be additional questions added to this second questionnaire based upon additional questions suggested by the panel during the first round of questioning. New questions may also be based around new themes raised within the free text given in the first round of questioning. You will be given 2 weeks to complete and submit this questionnaire with your considered responses.

These new responses will also be collated and evaluated by the research team. Overall data will be presented and reported within a final research report. You will receive a copy of this report. We anticipate that a final published research paper may also arise as a result of this research. You will also receive a copy of such a paper.

There is a risk that you may experience psychological distress as a result of participating in this study due to the nature of its sensitive topic. We would urge you to access support from your named healthcare professional or access alternative support here: (www.healthystaff4healthypatients.wordpress.com) should you be affected by any of the content put forward within this research study.

We would also urge you to take the recommended health and safety precautions whilst working in a desk based environment whilst completing the questionnaires involved.

The benefit of taking part in this study is that you will become a part of new research which explores the concept of a new online intervention designed to support midwives in psychological distress. Should this be developed, it will be evidence based as a result of your participation, making it potentially more suitable for use and of worth to the wider community.

We invite you to remain informed about the development of any subsequent online intervention designed to support midwives in psychological distress that may come as a result of this Delphi Study. However, you may remove yourself from this project at any time without giving reason.

Any data you give will be protected and secured confidentially by the research team. Other members of the panel will not know who else is participating. The public will not know who has participated, and any quotes reported within this literature will not identify the contributor. The data collected throughout this study will be kept securely for at least three years from the end of the project in line with Coventry University's policy on Principles and Standards of Conduct on the Governance of Applied Research.

Should you have any complaints, concerns or questions at any time, you may contact the primary researcher or the Director of Studies via the contact details below. You can also use these contact details to inform the research team of your withdrawal should you so wish.

Primary Researcher:

Sally Pezaro

Twitter: @SallyPezaro

Centre for Technology Enabled Health Research (CTEHR)

Faculty of Health & Life Sciences

Mile Lane

Coventry CV1 2NL

Tel: 07950035977

Email: pezaros@uni.coventry.ac.uk

Author:

Dr. Wendy Clyne PhD, BA (Hons)

Senior Research Fellow

FACULTY OF HEALTH AND LIFE SCIENCES

Coventry University

Priory Street

Coventry

CV1 5FB

Tel: 02477654636

Email: wendy.clyne@coventry.ac.uk

Should you not wish to participate, please ignore this correspondence.

Many thanks for your time and consideration

The Research Team.
