# Supplementary material for: Achieving Consensus in the Development of an Online Intervention Designed to Effectively Support Midwives in Work-Related Psychological Distress: Protocol for a Delphi Study
Source: JMIR Res Protoc. 2015 Sep 4;4(3):e107. doi: 10.2196/resprot.4766 (PMC4704889; doi:10.2196/resprot.4766)
Supplement: Multimedia Appendix 3 [file resprot_v4i3e107_app3.pdf]

Appendix 3: Informed Consent form

Achieving Consensus in the Development of an Online Intervention Designed to Effectively Support Midwives in Psychological Distress: A Delphi Study

This Delphi study forms a review of what should be included within an online intervention designed to support midwives in psychological distress, how this intervention should function, and how it should be used. The study will include 2 rounds of questioning, offered to a group of expert panelists. Questionnaire responses will be analysed in order to ascertain the direction in which the development of the online intervention should proceed. The aim is to form an expert consensus of opinion in this matter. You are invited to join the expert panel who will contribute to this study.

Please tick

1. I confirm that I have read and understood the participant information sheet for the above study and have had the opportunity to ask questions.

2. I understand that my participation is voluntary and that I am free to withdraw at any time without giving a reason

3. I understand that all the information I provide will be treated in confidence.

4. I understand that I also have the right to change my mind about participating in the study for a short period after the study has concluded (Deadline 1st December 2015).

5. I agree that my anonymised quotes can be used as part of the research project.

6. I agree to take part in the research project
