# Supplementary material for: Achieving Consensus in the Development of an Online Intervention Designed to Effectively Support Midwives in Work-Related Psychological Distress: Protocol for a Delphi Study
Source: JMIR Res Protoc. 2015 Sep 4;4(3):e107. doi: 10.2196/resprot.4766 (PMC4704889; doi:10.2196/resprot.4766)
Supplement: Multimedia Appendix 4 [file resprot_v4i3e107_app4.pdf]

Appendix 4: Delphi Study Questions for Round 1

Achieving Consensus in how an Online Intervention Designed to Effectively Support Midwives in Psychological Distress should be developed: Delphi survey Initial questionnaire

Thank you for agreeing to participate in this Delphi survey which aims to decide how an Online Intervention Designed to Effectively Support Midwives in Psychological Distress should be developed. This survey should take you approximately 30 minutes to complete.

You have been chosen to participate within this study as an expert panelist due to your relevant expertise. Your opinion is a valuable one.

This survey forms a review of what should be included within an online intervention designed to support midwives in work related psychological distress, how this intervention should function, and how it should be used.

This questionnaire round is the first of 2 questioning rounds. Please answer all questions by rating how much, or how little you would prioritise each case put forward. You will have the opportunity to write about why you have given your rating for each question at each stage of the questionnaire.

Once we have received all responses from all expert panelists, we will collate and summarise the findings and formulate a second round of questioning based upon the comments provided and answers given. We will invite you to participate in this second round of questioning approximately 5 weeks after the first questionnaires are distributed.

Your participation in this study and your individual responses will be strictly confidential to the research team. Other panelists will not be able to see your responses. Any comments that are published will be anonymised.

If you feel that you have been affected by any of the questions within this study, please access this link for external support.
(www.healthystaff4healthypatients.wordpress.com/support)

Below we ask some questions about what should be prioritized in the development of an online intervention designed to support midwives in work-related psychological distress. Each question asks about platform users and service users.
Platform users are defined as midwives who are interacting with the platform, and the service users are defined as the general public, patients and families.

There are 20 questions in total, organized into the following categories:

-Ethical Inclusions

-Inclusions of Therapeutic Support

-Intervention Design and Practical Inclusions

For each question we would like you to choose a number that best represents your response. There are no right or wrong answers. It is your opinion we are interested in. You might also like to write a few words about your answer in the text box below each question.

Please begin when you are ready.

An online intervention designed to support midwives in work-related psychological distress should prioritise:

-Ethical Inclusions

1. Confidentiality for all platform users and service users in all matters of discussion.

1 – Not a priority	2 – Low priority	3 – Somewhat priority	4 – Neutral	5 – Moderate Priority	6 – High priority	7 – Essential priority

(Optional open text field) – Why did you choose this rating of priority?

(Optional open text field) - Additional Comments:

2. Anonymity for all platform users and service users in all matters of discussion.

1 – Not a priority	2 – Low priority	3 – Somewhat priority	4 – Neutral	5 – Moderate Priority	6 – High priority	7 – Essential priority

(Optional open text field) – Why did you choose this rating of priority?

(Optional open text field) - Additional Comments:

3. Amnesty for all platform users in that they will not be referred to any law enforcement agencies, their employer or regulatory body for either disciplinary or investigative proceedings in any case.

1 – Not a priority	2 – Low priority	3 – Somewhat priority	4 – Neutral	5 – Moderate Priority	6 – High priority	7 – Essential priority

(Optional open text field) – Why did you choose this rating of priority?

(Optional open text field) - Additional Comments:

4. Prompting platform users automatically to remind them of their responsibilities to their professional codes of conduct.

1 – Not a priority	2 – Low priority	3 – Somewhat priority	4 – Neutral	5 – Moderate Priority	6 – High priority	7 – Essential priority

(Optional open text field) – Why did you choose this rating of priority?

(Optional open text field) - Additional Comments:

5. Prompting platform users automatically to seek help, by signposting them to appropriate support.

1 – Not a priority	2 – Low priority	3 – Somewhat priority	4 – Neutral	5 – Moderate Priority	6 – High priority	7 – Essential priority

(Optional open text field) – Why did you choose this rating of priority?

(Optional open text field) - Additional Comments:

- Inclusions of Therapeutic Support

6. The inclusion of web based videos, multimedia resources and tutorials which explore topics around psychological distress.

1 – Not a priority	2 – Low priority	3 – Somewhat priority	4 – Neutral	5 – Moderate Priority	6 – High priority	7 – Essential priority

(Optional open text field) – Why did you choose this rating of priority?

(Optional open text field) - Additional Comments:

7. The inclusion of informative multimedia designed to assist midwives to recognise the signs and symptoms of psychological distress.

1 – Not a priority	2 – Low priority	3 – Somewhat priority	4 – Neutral	5 – Moderate Priority	6 – High priority	7 – Essential priority

(Optional open text field) – Why did you choose this rating of priority?

(Optional open text field) - Additional Comments:

8. The inclusion of multimedia resources which disseminate self-care techniques

1 – Not a priority	2 – Low priority	3 – Somewhat priority	4 – Neutral	5 – Moderate Priority	6 – High priority	7 – Essential priority

(Optional open text field) – Why did you choose this rating of priority?

(Optional open text field) - Additional Comments:

9. The inclusion of multimedia resources which disseminate relaxation techniques

1 – Not a priority	2 – Low priority	3 – Somewhat priority	4 – Neutral	5 – Moderate Priority	6 – High priority	7 – Essential priority

(Optional open text field) – Why did you choose this rating of priority?

(Optional open text field) - Additional Comments:

10. The inclusion of information designed to inform midwives where they can access alternative help and support.

1 – Not a priority	2 – Low priority	3 – Somewhat priority	4 – Neutral	5 – Moderate Priority	6 – High priority	7 – Essential priority

(Optional open text field) – Why did you choose this rating of priority?

(Optional open text field) - Additional Comments:

11. The inclusion of information designed to inform midwives as to where they can access legal help and advice.

1 – Not a priority	2 – Low priority	3 – Somewhat priority	4 – Neutral	5 – Moderate Priority	6 – High priority	7 – Essential priority

(Optional open text field) – Why did you choose this rating of priority?

(Optional open text field) - Additional Comments:

12. Giving platform users the ability to share extended personal experiences for other platform users to read.

1 – Not a priority	2 – Low priority	3 – Somewhat priority	4 – Neutral	5 – Moderate Priority	6 – High priority	7 – Essential priority

(Optional open text field) – Why did you choose this rating of priority?

(Optional open text field) - Additional Comments:

13. The inclusion of a web based peer to peer discussion chat room.

1 – Not a priority	2 – Low priority	3 – Somewhat priority	4 – Neutral	5 – Moderate Priority	6 – High priority	7 – Essential priority

(Optional open text field) – Why did you choose this rating of priority?

(Optional open text field) - Additional Comments:

14. The inclusion of mindfulness tutorials and multimedia resources.

1 – Not a priority	2 – Low priority	3 – Somewhat priority	4 – Neutral	5 – Moderate Priority	6 – High priority	7 – Essential priority

(Optional open text field) – Why did you choose this rating of priority?

(Optional open text field) - Additional Comments:

15. The inclusion of Cognitive behavioural Therapy (CBT) tutorials and multimedia resources.

1 – Not a priority	2 – Low priority	3 – Somewhat priority	4 – Neutral	5 – Moderate Priority	6 – High priority	7 – Essential priority

(Optional open text field) – Why did you choose this rating of priority?

(Optional open text field) - Additional Comments:

16. Giving platform users the ability to communicate any work or home based subjects of distress.

1 – Not a priority	2 – Low priority	3 – Somewhat priority	4 – Neutral	5 – Moderate Priority	6 – High priority	7 – Essential priority

(Optional open text field) – Why did you choose this rating of priority?

(Optional open text field) - Additional Comments:

- Intervention Design and Practical Inclusions

17. An interface which does not resemble NHS, employer or other generic healthcare platforms.

1 – Not a priority	2 – Low priority	3 – Somewhat priority	4 – Neutral	5 – Moderate Priority	6 – High priority	7 – Essential priority

(Optional open text field) – Why did you choose this rating of priority?

(Optional open text field) - Additional Comments:

18. A simple, anonymised email login procedure which allows for continued contact and reminders which may prompt further platform usage.

1 – Not a priority	2 – Low priority	3 – Somewhat priority	4 – Neutral	5 – Moderate Priority	6 – High priority	7 – Essential priority

(Optional open text field) – Why did you choose this rating of priority?

(Optional open text field) - Additional Comments:

19. An automated moderating system where 'key words' would automatically initiate a moderated response.

1 – Not a priority	2 – Low priority	3 – Somewhat priority	4 – Neutral	5 – Moderate Priority	6 – High priority	7 – Essential priority

(Optional open text field) – Why did you choose this rating of priority?

(Optional open text field) - Additional Comments:

20. Mobile device compatibility for platform users

1 – Not a priority	2 – Low priority	3 – Somewhat priority	4 – Neutral	5 – Moderate Priority	6 – High priority	7 – Essential priority

(Optional open text field) – Why did you choose this rating of priority?

(Optional open text field) - Additional Comments:

(Optional open text field available for round 1 only) – Are there any further questions you would like to subject to an expert panel priority rating during the next round of questioning?
